# Can we offer additional BCG therapy for three-month BCG refractory high grade/T1, Tis bladder cancer patients?

**DOI:** 10.1080/2090598X.2023.2190687

**Published:** 2023-03-21

**Authors:** Amr A. Elsawy, Mahmoud Laymon, Islam Mansour, Ahmed Elghareeb, Ahmed Harraz

**Affiliations:** Urology Department, Urology and Nephrology Center, Mansoura University, Mansoura, Egypt

**Keywords:** Bladder cancer, BCG refractory, three-month cystoscopy, recurrence, progression

## Abstract

**Background:**

We lack tools to predict treatment and survival outcomes in patients receiving additional BCG therapy as a bladder-preserving therapy in high grade/T1, Tis NMIBC patients who showed persistent/recurrent tumors at three-month follow-up.

**Objectives:**

To assess the predictors of additional BCG response in patients who experienced persistent/recurrent tumors at three-month follow-up after BCG induction.

**Patients and methods:**

We retrospectively analyzed database for NMIBC. Between 2000 and 2019, 231 patients with high-grade T1/Tis NMIBC showed persistent/recurrent tumors at 3-month after BCG-induction, refused or were unfit to radical cystectomy (RC) and were offered additional intravesical BCG as bladder-preserving treatment. Predictors of the outcome after additional BCG were studied using univariate and multivariate logistic regression analysis. Kaplan Meier curve was utilized to estimate the recurrence-free survival (RFS) and progression-free survival (PFS). COX regression analysis was performed to identify independent predictors or RFS and PFS.

**Results:**

During a median (range) of 148 (24–224) months, poor response to additional BCG (tumor recurrence and/or progression) was noted in 112 (48.5%) patients. On multivariate logistic regression analysis, 3-month tumor features (persistent T stage, persistent grade and persistent/new CIS) significantly predicted poor response to additional BCG (OR: 3.4, 95%CI: 1.3–10.8, p = 0.021, OR: 2.1, 95%CI: 1.1–4.1, p = 0.02 and OR: 16.6, 95%CI: 4.5–109, *p*=<0.001, respectively). The mean RFS was 26 (9–152) months with identified 3-month tumor features (persistent T stage and persistent/new CIS) as independent predictors of RFS (HR = 11.5, 95%CI = 2.7–48.3, p = 0.001 and HR = 2.5, 95%CI = 1.5–4.1, *p*=<0.001, respectively) on multivariate COX regression analysis. In addition, 3-month tumor features (persistent/new CIS, non-papillary shape and bladder neck involvement) were identified to significantly predict PFS (HR = 6.2, 95%CI = 3.4–11.5, *p*=<0.001 and HR = 2.3, 95%CI = 1.3–4.3 p = 0.001 and HR = 2.1, 95%CI = 1.2–3.8, *p*=<0.005, respectively).

**Conclusions:**

Three-month tumor features could be utilized as a tool to predict treatment outcomes and survival benefits when additional intravesical BCG is utilized as a bladder-preserving treatment in patients with recurrent/persistent tumors at three-month follow-up.

## Introduction

After complete transurethral resection of bladder tumor (TURBT), intravesical immunotherapy with bacillus Calmette-Guérin (BCG) is the most effective adjuvant treatment in high-risk non-muscle invasive bladder cancer (NMIBC) [1]. The response to BCG therapy is widely variable with recurrence and progression rates reported in 32% to 43% and 9.5% to 14%, respectively [2]. Noteworthy, half of the patients who received adjuvant BCG therapy will eventually fail to achieve a sustained tumor-free status during long-term follow-up [3].

Currently, early radical cystectomy (RC) is offered as the standard of care in patients with BCG failure, several studies have demonstrated significant survival benefits when RC is performed within 24 months after the initial diagnosis [4]. Thus, patients’ survival could be augmented by early identification of those at high probability of BCG failure and direct them to optimal management; early RC [5]. Even, recurrent non-progressive disease exhausts health care systems with financial burdens on health care systems and is associated with patients’ morbidity with multiple procedures.

Despite superior survival benefits of RC in BCG-unresponsive patients, patients’ reluctance or unfitness for surgery constrains the clinicians for more exhaustion in bladder-preserving modalities as additional intravesical BCG, intravesical/systemic chemotherapy (Valrubicin, Gemcitabine or Docetaxel), intravesical photodynamic therapy (PDT) or intravesical immunotherapy with interferon-alpha [6].

Unavailability and lack of evidence for most of these therapies make the practitioners obliged to utilize additional intravesical BCG (second induction or proceed to maintenance) for patients who recur after initial response or those with persistent disease after first induction [7]. Prediction of response to this additional regimen of BCG is considered an important step whether in patients’ counseling or in the era of BCG shortage at which BCG should be prioritize for those with expected good response.

Many previous studies have investigated the different predictors of initial BCG response (Clinical/Pathological/Immune/Molecular) [8]. However, clinical-pathological predictors are still the simplest and most reliable tool in clinical practice to predict BCG response when compared to the novel exciting biomarkers which still lack adequate validation [8].

Noted tumor at 3-month check cystoscopy after BCG induction was shown to be a poor prognostic indicator for BCG response in high-risk NMIBC [9,10]. Grade 3 T1 recurrence at 3-month cystoscopy was previously evaluated as significant predictors of poor BCG response [11,12]. However, the impact of other clinical-pathological features of 3-month recurrence is not previously studied.

In this study, we aimed to study the predictors of additional BCG response in high grade/T1, Tis NMIBC who experienced primary BCG refractory response (recurrent/persistent tumor at 3-months) and were offered additional BCG therapy as a bladder-preserving treatment.

## Patients and methods

### Study population and eligibility criteria

After approval of Institutional Review Board for this study, database for NMIBC at our institute was reviewed for patients who underwent TURBT for high-risk NMIBC and completed 6 weekly doses of BCG induction from the period 2000 to 2019 and had experienced tumor recurrence at 3-month check cystoscopy.

We excluded patients with unavailable initial pathology for review and those who did not complete at least two years of follow-up from the 3-month cystoscopy. All pathology findings were re-checked by expert uro-pathologists to confirm tumor characteristics (stage according to TNM system and grade according to the1973 and 2004 World Health Organization grading classification system).

### Primary TURBT procedures and post-procedural strategy

During the aforementioned study period, all patients presenting for evaluation as bladder cancer were managed by endoscopic assessment with examination under anesthesia. Complete resection was achieved for all visible tumors. Immediate intravesical instillation of doxuribicin was routinely applied in all cases (unless contraindicated) within 24 hours after resection. High-risk NMIBC patients (T1, G3/high grade or CIS) underwent re-staging biopsy 4–6 weeks from the primary procedure after 2006 (and on case-by-case basis before 2006).

Patients usually started BCG induction 2–4 weeks after TURBT. Thereafter, all patients were evaluated by check cystoscopy 6 weeks after the last induction BCG dose. Patients with persistent/recurrent tumor (as confirmed by inpatient TURBT) were offered different treatment options including RC (preferably) or bladder preserving therapy using additional intravesical BCG (second induction followed by maintenance regimen according to the Southwest Oncology Group regimen [13]).

### Patients with 3-month recurrence/persistent tumors who received additional intravesical BCG; Follow-up

Patients who showed 3-month recurrence/tumor persistence and received additional intravesical BCG (either due to refusing or unfitness to RC) were followed-up every three months by cystoscopy and cytology with upper tract contrast imaging as clinically indicated.

### Study endpoints

In our study, further disease recurrence was defined as cystoscopic and pathological evidence of the disease, while, disease progression was defined as development of muscle invasive/nodal disease or distant metastasis. The primary endpoint included poor response to additional BCG which was defined as BCG-refractory and early BCG-relapsing tumor, i.e. within 6 months for high-grade papillary tumor and within 12 months for CIS [14].

### Statistical analysis

For categorical variables, Chi-square and Fisher’s exact tests were used for comparison whenever appropriate. Differences in continuous variables were evaluated using the Mann-Whitney U-tests. Kaplan-Meier curve with the log-rank test was used to compare recurrence and progression-free survivals (RFS and PFS) between both groups. Univariate and multivariate Cox regression analyses were processed to assess the impact of clinical and pathological data on RFS and PFS. Statistical analysis was performed using R programming language version 3.6.3 using the appropriate packages. P-value less 0.05 was considered statistically significant.

## Results

### Entire cohort

**Figure 1.**
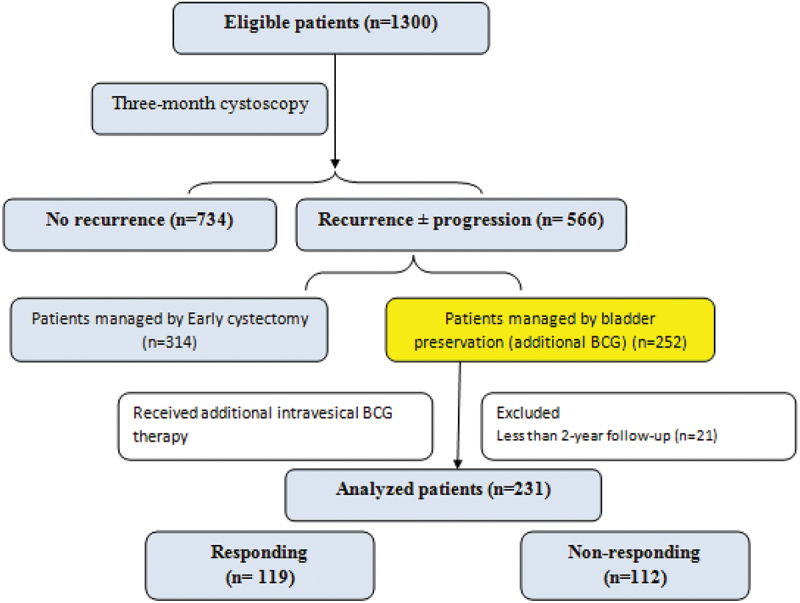
Study flow chart.

During the study period, 1300 patients were eligible for evaluation of their 3-month cystoscopy result after BCG induction. Of them, 566 (43.5%) patients showed recurrence ± progression as illustrated in study flow chart (Figure 1). Bladder preservation therapy using additional BCG instillation was decided in 252 patients, those who completed at least 24 months follow-up (231patients) were included in the final analysis.

The baseline clinical and pathological criteria of patients receiving additional BCG therapy after identified 3-month recurrence are shown in Table 1. Patients were followed-up accordingly for a median (range) of 148 (24–224) months. Poor response to additional BCG was noted in 112 (48.5%) patients (Figure 1) .

**Table 1.** Clinico-pathological characteristics of patients receiving additional BCG therapy after identified 3-month recurrence.

Variable	Value		
Age (year)		59.2 ± 10.4	
Gender			
Male		200 (86.6%)	
Female		31 (13.4%)	
Denovo vs. recurrent NMIBC			
Denovo		133 (57.6%)	
Recurrent		98 (42.4%)	
Initial tumor/s features		**Three-month tumor features**	**Change pattern of three-monthrecurrence**
Tumor number			
Single 83 (35.9%) Multiple 148 (64.1%)		Single 115 (49.8%) Multiple 116 (50.2%)	Regressed 64 (27.7%) Persistent 167 (72.3%)
T stage			
Ta 0 (0%) T1 209 (90.5%) Tis 22 (9.5%)		Ta 37 (16%) T1 188 (81.4%) Tis 6 (2.6%)	Regressed 37 (16%) Persistent 194 (84%)
Grade1973			
G1 0 (0%) G2 154 (66.7%) G3 77 (33.3%)		G1 26 (11.3%) G2 134 (58%) G3 71 (30.7%)	Regressed 55 (23.8%) Persistent 176 (76.2%)
Size			
<3 cm 109 (47.2%) > 3 cm 122 (52.8%)		<3 cm 157 (68%) > 3 cm 74 (32%)	Regressed 80 (34.6%) Progressed 151 (65.4%)
CIS			
No 209 (90.5%) Yes 22 (9.5%)		No 207 (89.6%) Yes 24 (10.4%)	No/regressed 207 (89.6%) New/persistent 24 (10.4%)
		**Shape**	
		Papillary 184 (79.7%) Non papillary* (flat, nodular, suspicious area) 47 (20.3%)	
		**Site**	
		Not including BN 165 (71.4%)	
		Including BN 66 (28.6%)	

### Clinico-pathological features of patients receiving additional BCG

The baseline clinical and pathological criteria of patients receiving additional BCG therapy are shown in Table 1. The change pattern of pathological features between the primary tumor and 3-month recurrence was assessed and defined as (persistent or regressed/regressed or progressed) (Table 1).

### Predictors of the outcomes after additional BCG

Poor response to additional BCG was identified in 112 (48.5%) patients. Three-month tumor features (persistent T stage, persistent grade, persistent/new CIS and tumors with bladder neck involvement-BNI-) were shown to predict the outcome of additional BCG. Only three factors persistent T stage, persistent grade and persistent/new CIS maintained their independent relationship on multivariate analysis (OR: 3.4, 95%CI: 1.3–10.8, p = 0.021, OR: 2.1, 95%CI: 1.1–4.1, p = 0.02 and OR: 16.6, 95%CI: 4.5–109, *p*=<0.001, respectively) (Table 2).

**Table 2.** Univariate and multivariate logistic regression analysis of predictors of additional BCG response.

Univariate			
	No	Yes	p-value
Age	58.6 ± 11.2	59.9 ± 9.5	0.347
Gender			0.173
Male	99 (49.5%)	101 (50.5%)	
Female	20 (64.52%)	11 (35.48%)	
Denovo vs. recurrent NMIBC			0.793
Denovo	70 (52.63%)	63 (47.37%)	
Recurrent	49 (50%)	49 (50%)	
Three-month tumor change pattern compared to initial tumor			
CIS			0.003
No/regressed	114 (55.07%)	93 (44.93%)	
New/persistent	5 (20.83%)	19 (79.17%)	
Grade			0.002
Regressed	39 (70.91%)	16 (29.09%)	
Persistent	80 (45.45%)	96 (54.55%)	
No			0.307
Regressed1	29 (45.31%)	35 (54.69%)	
Persistent	90 (53.89%)	77 (46.11%)	
Size			0.527
Regressed	44 (55%)	36 (45%)	
Progressed	75 (49.67%)	76 (50.33%)	
T stage			<0.001
Regressed3	35 (94.59%)	2 (5.41%)	
Persistent1	84 (43.3%)	110 (56.7%)	
Three-month tumor shape			0.375
Papillary	98 (53.26%)	86 (46.74%)	
Non papillary (flat, nodular, suspicious area)	21 (44.68%)	26 (55.32%)	
Three-month tumor site			0.013
Outside BN	94 (56.97%)	71 (43.03%)	
BN involvement	25 (37.88%)	41 (62.12%)	
MULTIVARIATE			
	**OR**	**95%CI**	
CIS: New/persistent	16.6	(4.5–109.2)	<0.001
Grade: Persistent	2.1	(1.1–4.1)	0.021
T stage ‘Persistent	3.4	(1.3–10.8)	0.021
Three-month tumor site;` BN involvement	1.3	(0.6–3)	0.498

### Predictors of recurrence-free survival (RFS)

**Figure 2.**
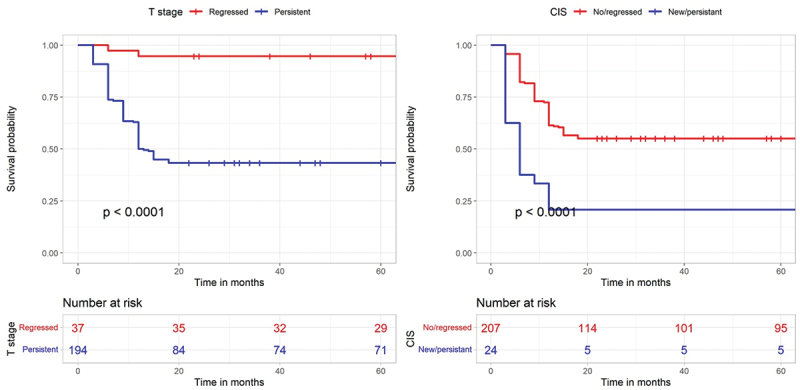
Kaplan-Meyer survival curves; Recurrence-free survival .

During follow-up, tumor recurrence was observed in 112 (48.5%) patients. On multivariate COX regression analysis, 3-month recurrence features (persistent T stage and persistent/new CIS) were identified to significantly predict RFS (HR = 11.5, 95%CI = 2.7–48.3, p = 0.001 and HR = 2.5, 95%CI = 1.5–4.1, *p*=<0.001, respectively) (Table 3). The mean RFS was 26 (9–152) months. Survival curves are shown in Figure 2.

**Table 3.** Univariate and multivariate Cox regression analysis of recurrence and progression-free survivals.

Univariate							
	Recurrence-free survival				Progression-free survival		
	Hazards ratio	95%CI		p-value	Hazards ratio	95%CI	p-value
Age	1	(1–1)		0.529	1	(1–1)	0.525
Gender; female	0.6	(0.3–1.1)		0.107	0.6	(0.2–1.6)	0.266
Denovo vs. recurrent NMIBC; recurrent	1.1	(0.7–1.5)		0.742	1.3	(0.7–2.3)	0.424
Three-month recurrence change pattern compared to initial tumor							
CIS; New/persistent	3.4	(2.1–5.6)		<0.001	8.5	(4.7–15.6)	<0.001
Grade; Persistent	2.1	(1.3–3.6)		0.005	1.6	(0.7–3.4)	0.237
Number; Persistent	0.7	(0.5–1.1)		0.143	0.7	(0.4–1.4)	0.315
Size; Progressed	1.1	(0.7–1.6)		0.695	0.7	(0.4–1.3)	0.261
T stage; Persistent	15	(3.7–60.6)		<0.001	0.9	(0.8–1.5)	0.345
Three-month tumor shape; non papillary (flat, nodular, suspicious area)	1.4	(0.9–2.2)		0.109	3.2	(1.8–5.8)	<0.001
Three-month tumor site; BN involvement	1.6	(1.1–2.4)		0.014	2.2	(1.3–4)	0.006
MULTIVARIATE							
CIS; New/persistent	2.5		(1.5–4.1)	<0.001	6.2	(3.4–11.5)	<0.001
Grade; Persistent	1.1		(0.6–1.9)	0.712			
T stage;` Persistent	11.5		(2.7–48.3)	0.001			
Three-month tumor site; `BN involvement	1.4		(1–2.1)	0.061	2.1	(1.2–3.8)	0.005
Three-month tumor shape; non papillary (flat, nodular, suspicious area)					2.3	(1.3–4.3)	0.001

### Predictors of progression-free survival (PFS)

**Figure 3.**
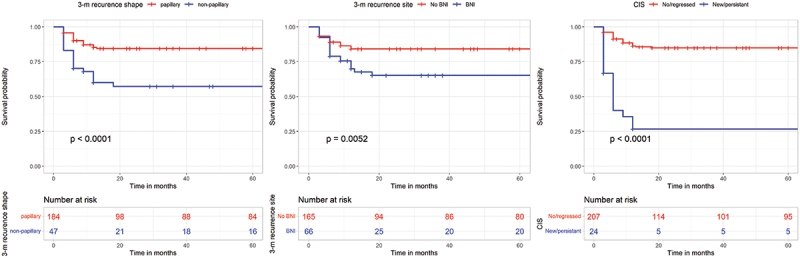
Kaplan-Meyer survival curves; Progression-free survival .

During follow-up, tumor progression was defined as development of muscle invasive disease. Tumor progression was observed in 46 (19.9%) patients. On multivariate Cox regression analysis, 3-month recurrence features (persistent/new CIS, non-papillary shape and bladder neck involvement) were identified to significantly predict PFS (HR = 6.2, 95%CI = 3.4–11.5, *p*=<0.001 and HR = 2.3, 95% CI = 1.3–4.3 p = 0.001 and HR = 2.1, 95%CI = 1.2–3.8, *p*=<0.005, respectively) (Table 3). The mean RFS was 26 (9–152) months. Survival curves are shown in Figure 3.

## Discussion

Intravesical BCG has been established as a standard adjuvant treatment after TURBT for high-risk NMIBC [15–17]. Treatment response to intravesical BCG is primarily evaluated after completing the 6 weeks induction regimen by 3-month check cystoscopy and cytology [18]. In approximately 50% of cases, BCG treatment eventually fails, and half of them experience recurrence in the first 6 months after complete induction with/out one maintenance course of BCG (BCG refractory) [3].

Treatment options for patients with identified early recurrence (at 3-month cystoscopy) is still a matter of discussion around either early RC, additional BCG (re-induction or proceed to maintenance), other intravesical/systemic therapeutic agents, radiation therapy or a clinical trial [19]. Many previous reports have shown that early RC was the standard treatment in patients with T1 or high grade recurrent tumors at 3-month cystoscopy [20]. Nevertheless, in patients who refuse/are unfit to RC due to co-morbidities, bladder-preserving strategies should be counseled [20].

It is worthy to note that the only FDA-approved therapeutic option for primary BCG failure are intravesical pembrolizumab and valrubicin. However, this has conveyed minor modification in practice as valrubicin effectiveness has been argued by many professionals as the registration trial was questionable and Pembrolizumab remains a costly, difficult to access with a role yet to be determined [21].

Despite its proven inferiority to RC in patients who experienced initial BCG-unresponsiveness, there is lack in the literature about the precise survival benefits of additional BCG therapy in such patients. Moreover, the current dilemma of BCG shortage has obliged the health care providers to prioritize the patients with favorable response of BCG. Therefore, prompt characterization of patients with anticipated additional BCG-unresponsiveness is warranted for optimal counseling and saving of health care costs [22].

With respect to BCG-failure, most of the definitions are either anecdotal or through a panel consensus with lack of a data-driven evaluation. In the real practice, 3-month cystoscopy recurrence after completed BCG induction course is usually defined by practitioners as a surrogate marker of initial BCG-unresponsiveness and critical distinction to do in the patients’ management either by RC or different bladder-preserving options [23].

In our cohort, among patients who experienced 3-month recurrent/persistent tumors, approximately half of them chose RC. The other group was managed by additional BCG intravesical therapy (being the only adapted bladder-preserving option after initial BCG unresponsiveness at our institute). Catto et al. had reported an acceptance rate; 25% for RC in high-risk NMIBC when they were offered allocated treatment (RC vs. BCG) [24]. This discrepancy in the acceptance rate could be justified by the nature of our institute as a tertiary referral center for bladder cancer patients with initial diagnosis made at our hospital excluding the multiple distracting opinions for the patients.

In our study, we hypothesized that both clinico-pathological features of three-month recurrence and its pattern when compared to primary tumor characteristics can be utilized as an indicator of disease severity and provide a predictive instrument not only of additional BCG-unresponsive, but also the survival features (RFS and PFS).

In the study cohort, new/persistent CIS at 3-month cystoscopy recurrence was identified as an independent predictor of additional-BCG unresponsiveness. Moreover, these pathological features adversely compromise the survival outcomes. Our finding copes with what have been previously reported by Tang and colleagues who concluded that presence of CIS at 3–6 month after induction BCG can adversely affect the treatment and survival outcomes [25]. Additionally, Herr et al. had demonstrated the compromised PFS in patient with CIS at 3-month recurrence when received additional BCG [26].

As regard to T stage of 3-month recurrence, persistence T stage was noted to inversely predict the additional BCG response. Furthermore, persistent T stage significantly compromised RFS. In the same way, a previous study by Chinedu and colleagues, T stage of 3-month recurrence (Ta versus T1), was shown to influence treatment response after additional BCG therapy for BCG refractory patients [12], 5-year disease progression was identified in only 5% of patients with Ta recurrence. Similarly, Solsona et al. demonstrated the impact of Ta stage in 3-month recurrence on disease progression when additional BCG was utilized (5-year disease progression; 10%) [11].

Moreover, persistent grade of 3-month recurrence was identified as another major predictor of response to additional BCG therapy. Low-grade tumor at 3-month recurrence did not demonstrate noteworthy events when associated with Ta stage; in agreement findings previously shown by Chinedu et al [12] and Herr et al [27]. On the other hand, persistent grade did not maintain its significance (observed in the univariate analysis) in the multivariate analysis of RFS.

BNI at 3-month cystoscopy recurrence was identified in our cohort to significantly compromise the PFS. This comes in hand with what has been previously concluded by Kobayashi and associates about the prognostic impact of BNI on disease progression in primary NMIBC [28]. Additionally, non-papillary shape of 3-month recurrence independently associated with disease progression. Non-papillary morphology of NMIBC was previously demonstrated by Park et al. as a predictor of cancer progression [29].

Although the noted implications of 3-month recurrence features (T stage, CIS, grade, shape and BNI) on treatment response and survival outcomes of additional BCG therapy after primary BCG-unresponsiveness, we did not observe a significant impact of 3-month recurrence number. On the contrary, tumor number has been previously studied as predictor of BCG response. Steinberg et al have shown that patients with≥2 prior BCG courses revealed worse outcomes with multifocal tumor, while those with≤1 prior BCG course showed equivalent outcomes [23]. Given the influence of tumor number on treatment outcomes, it can be argued that some of these tumors may be overlooked from the primary TURBT and not a true recurrence, as well as the specific stage, grade and size of each tumor, remains incompletely evaluated.

In this study, we provide the clinicians with a useful predictive tool for treatment responsiveness and survival outcomes of additional BCG therapy after initial BCG-unresponsiveness. The findings of our analysis should be cautiously interpreted due to several limitations. Above all, there might be inherent bias resulting from its retrospective nature. Other potential prognostic factors that we did not include in our data, such as preoperative urine cytology, repeat biopsy, and detrusor muscle inclusion, variant histology and lympho-vascular invasion (LVI) which were suggested as significant predictor of BCG therapy in the different studies [24,25,26], could be further studied to augment their utilization in clinical practice.

Given that our study consisted of TURBT procedures done by multiple surgeons, the outcome could be confounded by the surgeons’ experience with TURBT. In addition, owing to the limitation of the retrospective analysis, it is difficult to identify the exact information about previously administered regimen of intravesical chemotherapy/immunotherapytherapy in patients with history of NMIBC. Finally, although we identified valuableinformation about additional BCG therapy in patients who experienced recurrenceat 3-month recurrence, further prospective studies are warranted to promote our findings.

## Conclusions

Features of 3-month cystoscopy recurrence after BCG induction significantly predicted treatment outcome and survival benefits after additional BCG therapy when utilized as a bladder-preserving treatment in patients unfit/refusing RC.

## Abbreviations

BNI Bladder neck involvement

BCG Bacillus Calmette-Guérin

CIS Carcinoma in situ

NMIBC Non muscle invasive bladder cancer

PFS Progression-free survival

RC Radical cystectomy

RFS Recurrence-free survival

TURBT Transurethral resection of bladder tumor

## Clinical trials registration

clinicaltrials.gov ID: NCT04319263

## Descriptive key message

The current study aimed at investigating the predictors of additional intravesical BCG therapy as a bladder-preserving treatment in high grade/T1, Tis NMIBC with three-month persistent/recurrent tumors.
